# Supplementary material for: The microbiome biomarkers of pregnant women’s vaginal area predict preterm prelabor rupture in Western China
Source: Front Cell Infect Microbiol. 2024 Oct 31;14:1471027. doi: 10.3389/fcimb.2024.1471027 (PMC11560878; doi:10.3389/fcimb.2024.1471027)
Supplement: Supplementary file 1 [file DataSheet1.zip › compare_1/Community/Pieplot/family/sample/C17.family.pieplot.pdf]

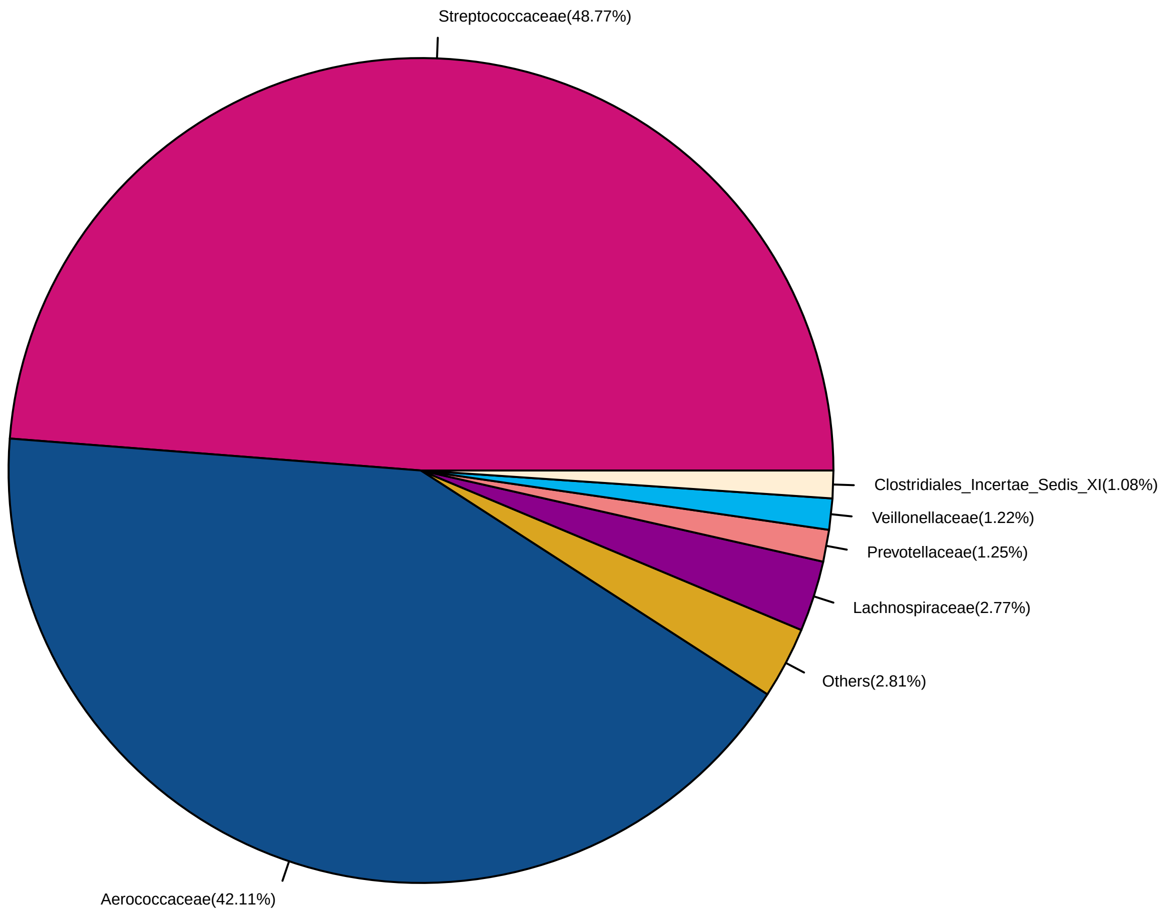
Streptococcaceae(48.77%)
Clostridiales_Incertae_Sedis_XI(1.08%)
Veillonellaceae(1.22%)
Prevotellaceae(1.25%)
Lachnospiraceae(2.77%)
Others(2.81%)
Aerococcaceae(42.11%)
